# MCLPMDA: A novel method for miRNA‐disease association prediction based on matrix completion and label propagation

**DOI:** 10.1111/jcmm.14048

**Published:** 2018-11-29

**Authors:** Sheng‐Peng Yu, Cheng Liang, Qiu Xiao, Guang‐Hui Li, Ping‐Jian Ding, Jia‐Wei Luo

**Affiliations:** ^1^ School of Information Science and Engineering Shandong Normal University Jinan China; ^2^ College of Information Science and Engineering Hunan Normal University Changsha China; ^3^ School of Information Engineering East China Jiaotong University Nanchang China; ^4^ College of Information Science and Engineering Hunan University Changsha China

**Keywords:** label propagation, matrix completion, miRNA‐disease association prediction

## Abstract

MiRNAs are a class of small non‐coding RNAs that are involved in the development and progression of various complex diseases. Great efforts have been made to discover potential associations between miRNAs and diseases recently. As experimental methods are in general expensive and time‐consuming, a large number of computational models have been developed to effectively predict reliable disease‐related miRNAs. However, the inherent noise and incompleteness in the existing biological datasets have inevitably limited the prediction accuracy of current computational models. To solve this issue, in this paper, we propose a novel method for miRNA‐disease association prediction based on matrix completion and label propagation. Specifically, our method first reconstructs a new miRNA/disease similarity matrix by matrix completion algorithm based on known experimentally verified miRNA‐disease associations and then utilizes the label propagation algorithm to reliably predict disease‐related miRNAs. As a result, MCLPMDA achieved comparable performance under different evaluation metrics and was capable of discovering greater number of true miRNA‐disease associations. Moreover, case study conducted on Breast Neoplasms further confirmed the prediction reliability of the proposed method. Taken together, the experimental results clearly demonstrated that MCLPMDA can serve as an effective and reliable tool for miRNA‐disease association prediction.

## 1 INTRODUCTION

MiRNAs are a class of small endogenous single‐stranded non‐coding RNAs (~22 nt RNAs).^1^, ^2^, ^3^, ^4^ Since the discovery of the first two miRNAs lin‐4 and let‐7, increasing evidences have indicated that miRNAs play vital roles in a variety of complex biological process, such as cell differentiation, proliferation, apoptosis and signal transduction. For instance, by performing the pathway enrichment analysis for targets of differentially expressed miRNAs recorded from databases, Calin and Croce demonstrated that the down‐regulation of the suppressor miR‐15a/miR‐16‐1 induces overexpression of BCL2 and possibly other genes that may be important for tumourigenesis, whereas the overexpression of oncogenic miR‐17‐92 cooperates with c‐Myc in stimulating proliferation.^5^ In addition, Ma et al. indicated that miR‐10b is highly expressed in metastatic breast cancer cells and positively regulates cell migration and invasion.^6^ Recently, Zhang et al. identified miRNA‐26a as a key regulon that inhibits progression and metastasis of c‐Myc/EZH2 double high advanced hepatocellular carcinoma.^7^ Besides, plenty of studies have indicated that miRNA mutations or misexpression are closely related with various human cancers and thus miRNAs could act as tumour suppressors and oncogenes.^8^, ^9^, ^10^ Therefore, prediction of potential miRNA‐disease associations makes an important contribution to understanding the molecular mechanism of disease pathogenesis and further promoting the level of treatment.

Traditional experimental methods such as qRT‐PCR^11^ and microarray profiling^12^ have been adopted to identify miRNA‐disease association predictions. Although reliable, experiment‐based methods are generally expensive and time‐consuming. With the rapid development of biotechnology, a vast amount of publicly available RNA‐related datasets have been released, which also provides great opportunities for uncovering potential associations between diseases and miRNAs by taking advantage of these data resources computationally. Recently, considerable efforts have been made to discover disease‐associated miRNAs based on the assumption that miRNAs with similar functions are tend to be associated with similar disease.^13^ Jiang et al. constructed a human phenome‐miRNAome functional association network and proposed the first computational model to infer the candidate disease‐related miRNAs based on the hypergeometric distribution scoring system. By testing the proposed model on 270 known experimentally verified miRNA‐disease associations, they achieved an accuracy of 0.758 in leave‐one‐out cross validation (LOOCV).^14^ They further proposed a weighted network‐based method to improve the calculation of concordance score between a specific miRNA and a given disease, and achieved an area under the receiver operating characteristic curve (AUC) value of 0.80 in global LOOCV.^15^ Nevertheless, the high false‐positive rate in miRNA target predictions severely limited the efficacy of Jiang's methods. By incorporating miRNA‐target interactions, disease‐gene associations and protein‐protein interactions, Shi et al. introduced a modified random walk algorithm with restart (RWR) to identify miRNA‐disease associations. As a result, their approach achieved satisfactory performance in identifying known cancer‐related miRNAs for nine human cancers with an AUC value of 0.713 and 0.913 in LOOCV framework.^16^ Similarly, Mørk et al. presented a miRNA‐Protein‐Disease network by integrating known miRNA‐protein associations and disease‐protein interactions to infer potential miRNAs associated with each investigated disease.^17^ Later, Xu et al. utilized known disease‐related protein‐coding genes to prioritize miRNAs‐disease associations according to context‐dependent miRNA‐target interactions and obtained an average overall prediction accuracy of 0.887 in cross‐validation tests.^18^ In contrast to previous methods, Xu's method does not depend on known disease‐related miRNAs. However, their method also suffers from the high false positive rates and false negative rates existed in the predicted miRNA‐target interactions. By integrating known associations, disease semantic similarity, miRNA functional similarity and Gaussian interaction profile kernel similarity, Chen et al. calculated a within‐score and a between score to gain an eventual confidence score for miRNA‐disease associations. Specifically, they obtained an AUC value of 0.8031 in LOOCV, which clearly demonstrated their improvement.^19^ Considering the fact that there are only very few known miRNA‐disease associations and many associations are “missing” in the known training database, Chen et al. introduced the concepts of “super‐miRNA” and “super‐disease” to enhance the miRNA similarity and disease similarity measures to infer disease‐related miRNAs.^20^ Specifically, their method could be applied to new diseases without any known associated miRNAs as well as new miRNAs without any known associated diseases. As a result, their method achieved reliable performance with AUCs of 0.9032, 0.8323 and 0.8970 in global LOOCV, local LOOCV and 5‐fold cross validation respectively.

In addition, machine learning‐based methods for predicting miRNA‐disease association have attracted widespread attention.^21^, ^22^, ^23^, ^24^, ^25^, ^26^ Chen et al. proposed a novel computational model based on heterogeneous graph inference for miRNA‐disease association prediction by integrating miRNA functional similarity, disease semantic similarity, kernel similarity of Gaussian interaction profile and experimentally validated miRNA‐disease associations into a heterogeneous network.^21^ Concretely, HGIMDA adopted an iterative process to find the optimal solutions based on global network similarity information, which led to superior performance over local network similarity‐based methods. HGIMDA obtained AUCs of 0.8781 and 0.8077 in terms of global and local LOOCV respectively. Xiao et al. developed a novel graph regularized non‐negative matrix factorization framework to simultaneously identify the potential associations for all diseases^22^ and their model was relatively robust to the noises in the datasets. As a result, Xiao's method achieved an AUC value of 0.869 based on LOOCV framework. Under the motivation to find out the deep representation of disease semantic similarity, miRNA functional similarity and known miRNA‐disease associations, Chen et al. proposed DRMDA to predict miRNA‐disease associations. The main advantage of the deep representation lies in that some noise within unprocessed data can be eliminated while features related with association can be clearly presented^23^. The AUCs achieved by DRMDA were 0.9177, 0.8339 and 0.9156 in global LOOCV, local LOOCV and 5‐fold cross validation respectively. Peng et al. proposed a novel computational model named NARRMDA to score and rank miRNAs for a given disease without known associations. NARRMDA combined a rating‐based recommendation algorithm and a negative‐aware algorithm to predict disease‐related miRNAs,^24^ and it achieved an AUC value of 0.8053 in LOOCV framework. Later, Chen et al. proposed a novel method called MKRMDA which automatically optimizes the multiple kernel combinations of both diseases and miRNAs.^25^ MKRMDA achieved remarkable AUCs of 0.904, 0.8446 and 0.8894 in global, local LOOCV and 5‐fold cross validation respectively. They further developed the first decision tree learning‐based model, EGBMMDA, to discover the potential miRNA‐disease associations by employing Extreme Gradient Boosting Machine.^26^ The experimental results indicated that EGBMMDA obtained AUC values of 0.9123, 0.8221 and 0.9048 in global, local LOOCV and 5‐fold cross validation respectively. Although effective, a common limitation of methods using machine learning schema mentioned above is that there are no validated negative samples for miRNA‐disease associations.

Recently, several path‐based methods taking advantage of network topological structures have been proposed to predict miRNA‐disease associations. Sun et al. proposed a method called NTSMDA which only utilizes the miRNA‐disease network topological similarity to predict disease‐associated miRNAs^27^ and achieved an AUC of 0.894 by using the LOOCV experiment. Chen et al. devised a method GIMDA based on graphlet interaction which was applied to analyse the relevance between two points.^28^ The AUCs of GIMDA in global, local LOOCV and 5‐fold cross validation turned out to be 0.9006 and 0.8455 and 0.8927 respectively. However, as NTSMDA and GIMDA strongly depends on network topological structure, they cannot be applied to diseases without any known associated miRNAs. You et al. first constructed a heterogeneous network and then proposed a model called PBMDA by performing a depth‐first search algorithm on the heterogeneous network to infer disease‐related miRNAs.^29^ In particular, PBMDA achieved reliable performance in the frameworks of both local and global LOOCV (AUCs of 0.8341 and 0.9169 respectively) and 5‐fold cross validation (average AUC of 0.9172). An obvious superiority of PBMDA compared with NTSMDA and GIMDA was that it can be applied to new diseases and new miRNAs, which greatly improved the practicability and reliability of PBMDA. Recently, Chen et al. proposed NDAMDA based on network distance to predict miRNA‐disease associations. NDAMDA not only considered the direct network distance between two miRNAs or diseases but also took their respective mean network distances to all other miRNAs or diseases into account.^30^ The reliable performance of NDAMDA was certified by the AUCs of 0.8920, 0.8062 and 0.8935 obtained in global LOOCV, local LOOCV and 5‐fold cross validation respectively.

Although existing methods have made great contributions to uncover disease‐related miRNAs, there are still some limitations that could be improved in many aspects. Therefore, in this paper, we develop a novel method for miRNA‐disease association prediction based on Matrix Completion and Label Propagation (MCLPMDA). An important innovation of MCLPMDA is that it leverages matrix completion algorithm to solve the problem of sparsity and incompletion, which greatly improves the prediction accuracy. To demonstrate the effectiveness of our proposed method, we apply different evaluation metrics to comprehensively measure the prediction performance. In addition, we compare our method with four state‐of‐the‐art methods and the results indicate that our method could achieve comparable performance. Moreover, the results of case study on Breast Neoplasms (BN) further verify the reliability and robustness of MCLPMDA. Together, all the results demonstrate that MCLPMDA can serve as an effective tool for discovering miRNA‐disease associations.

## 2 MATERIALS AND METHODS

### 2.1 Human miRNA‐disease associations

MiRNA‐disease associations were downloaded directly from the HMDD v2.0 which contains 5340 experimentally verified links between 495 miRNAs and 383 diseases.^31^ We used an adjacency matrix *DM* to describe the obtained miRNA‐disease associations. Concretely, the element *DM*(*i,j*) is 1 if disease *d*(*i*) is verified to be associated with miRNA *m*(*j*), and 0 otherwise. Therefore, the *i*‐th row of *DM* is a binary vector representing the associations between disease *d*(*i*) and each miRNA, while the *j*‐th column of *DM* represents the associations between miRNA *m*(*j*) and each disease.

### 2.2 MiRNA functional similarity

MiRNA functional similarity scores were computed based on the assumption that functionally similar miRNAs are more likely to connect with phenotypically similar disease.^32^, ^33^ In this paper, we downloaded the miRNA functional similarity scores directly from http://www.cuilab.cn/files/images/cuilab/misim.zip. We used matrix *FM* to denote the obtained miRNA functional similarity network, in which *FM*(*i,j*) indicates the similarity between miRNA *m*(*i*) and miRNA *m*(*j*).

### 2.3 Disease semantic similarity

Mesh database ( http://www.ncbi.nlm.nih.gov/) is a strict system for disease classification and is a credible dataset for effectively researching the association between different diseases. A disease can be described as a directed acyclic graph, *DAG* = (*D*,*T*(*D*),*E*(*D*)), where *T*(*D*) represents both node *D* and its ancestor nodes, and *E*(*D*) represents all direct edges connecting the parent nodes to child nodes. The contribution values of disease *d* to the semantic value of disease *D* can be calculated as follows:

(1) $$\left\{\begin{array}{cc} D_{D}\left(d\right)=1 & \text{if}\;d=D\\ D_{D}\left(d\right)=\max\left\{\Delta *D_{D}\left(d^{\prime }\right)\left|\right.d^{\prime }\in \text{childen of}\;d\right\} & \text{if}\;d\neq D \end{array}\right.$$

Here, ∆ is the semantic contribution factor and we set ∆=0.5 in this paper. For disease *D*, the contribution of itself is 1, while the contribution of another disease *d*_*j*_ decreases as the distance between *D* and *d*_*j*_ increases. Hence, the semantic value of disease *D* can be calculated according to the contribution of ancestor diseases and disease *D* itself:

(2) $$DV\left(D\right)=\sum_{t\in T(D)}D_{d}\left(t\right)$$

Then, the semantic similarity between disease *d*_*i*_ and disease *d*_*j*_ could be calculated as follows:

(3) $$S\left(d_{i},d_{j}\right)=\frac{\sum_{T\in T\left(d_{i}\right)\cap T\left(d_{j}\right)}\left(D_{d_{i}}\left(t\right)+D_{d_{j}}\left(t\right)\right)}{DV\left(d_{i}\right)+DV\left(d_{j}\right)}$$

According to Equation (3), we can construct an overall disease semantic similarity matrix *SD* where *SD*_*ij*_ represents the semantic similarity between disease *d*_*i*_ and disease *d*_*j*_.

### 2.4 Gaussian interaction profile kernel similarity for miRNAs and diseases

Based on the assumption that functional similar miRNAs tend to be associated with similar diseases and vice versa, we first constructed Gaussian interaction profile kernel similarity for miRNAs.^34^ Specifically, a binary vector *M*(*i*) representing the *i*‐th column of the adjacency matrix *DM* is considered as the interaction profiles of miRNA *m*(*i*). The Gaussian kernel similarity between miRNA *m*(*i*) and *m*(*j*) can then be calculated as follows:

(4) $$GM\left(m\left(i\right),m\left(j\right)\right)=\exp\left(-\gamma_{m}{∥M(i)-M(j)∥}^{2}\right)$$

where γ_*m*_ is a parameter to control the kernel bandwidth and it can be obtained by the following formula:

(5) $$\gamma_{m}=\frac{\delta_{m}}{\frac{1}{\mathrm{nm}}{\sum_{i=1}^{\mathrm{nm}}∥M(i)∥}^{2}}$$

where δ_*m*_ is a new bandwidth parameter and *nm* denotes the number of all the miRNAs. Similarly, the Gaussian interaction profile kernel similarity between disease *d*(*i*) and *d*(*j*) is calculated by:

(6) $$GD\left(d\left(i\right),d\left(j\right)\right)=\exp\left(-\gamma_{d}{∥D(i)-D(j)∥}^{2}\right)$$

(7) $$\gamma_{d}=\frac{\delta_{d}}{\frac{1}{\mathrm{nd}}{\sum_{i=1}^{\mathrm{nd}}\left|M(i)\right|}^{2}}$$

For simplicity, δ_*m*_ and δ_*d*_ were set to 1 according to previous studies.^32^, ^34^, ^35^, ^36^

### 2.5 MCLPMDA

**Figure 1:**
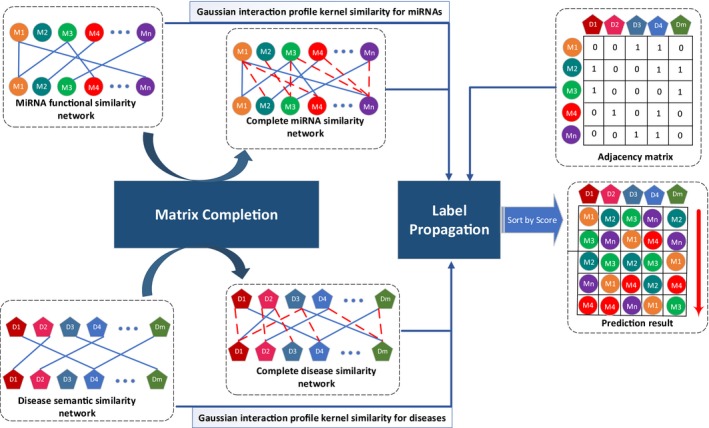
Flowchart of potential disease‐miRNA association prediction based on the computational model of MCLPMDA. Our algorithm mainly consists of three steps: (1) we construct a new miRNA similarity matrix as well as a disease similarity matrix based on matrix completion algorithm; (2) the two reconstructed similarity matrices are combined with Gaussian interaction profile kernel similarity for miRNAs and diseases respectively; (3) label propagation algorithm is conducted in both miRNA space and disease space to obtain the final prediction results

As mentioned above, due to the inherent noise in the current datasets, the obtained miRNA functional similarity matrix and disease semantic similarity matrix might be sparse and incomplete, which have greatly limited the prediction accuracy of existing methods. In this work, we developed a novel method named MCLPMDA to predict miRNA‐disease associations based on matrix completion and label propagation. MCLPMDA can be simply divided into three steps: firstly, we construct a new miRNA similarity matrix *CM* as well as a disease similarity matrix *CD* based on matrix completion algorithm. Secondly, we combine the two constructed similarity matrices with existing similarity information for miRNAs and diseases respectively. Thirdly, we conduct label propagation algorithm in both miRNA space and disease space to obtain the final prediction results. An overall workflow of MCLPMDA is illustrated in Figure 1.

#### 2.5.1 Matrix completion for miRNA and disease

The present data are often far from perfect, meaning that, a part of the dataset would be incorrect or missing.^37^ Therefore, an incomplete data matrix *D* can be decomposed into two parts. The first part is a linear combination of *D*, which is a low‐rank matrix and is essentially a projection from the noisy data *D* into a more refined or informative lower‐dimensional space. The second part is a noise data matrix separated from the original data matrix *D*. According to the above statement, *D* can be decomposed as follows:

(8) D=DR+N

Apparently, Equation (8) has infinite solutions. However, as we want *R* to be low‐rank and *N* to be sparse, we add nuclear norm or trace norm on *D* and adopt the ℓ_2,1_ norm to characterize the error term *N*. Specifically, we could obtain a low‐rank recovery matrix by solving the following convex optimization problem:

(9) $$\min_{R,N}{∥R∥}_{*}+\omega {∥N∥}_{2,1}s.t.\;D=DR+N$$

where ${∥R∥}_{*}=\sum_{k}\sigma_{k}$ (i.e., σ_*k*_ is the singular values of *D*) donates the nuclear norm of a matrix, ${∥N∥}_{2,1}=\sum_{i=1}^{n}\sqrt{\sum_{j=1}^{m}{\left(N_{\mathrm{ij}}\right)}^{2}}$ is the noise regularization term and ω is the positive weighting parameter to balance the weights of low‐rank matrix *R* and sparse matrix *N*.^38^ After obtaining the minimizer (R*, N*), we could use DR* (or *D* − N*) to obtain a low‐rank recovery matrix *CD*.

The optimization problem (9) is convex and can be solved in various ways, for example, accelerated proximal gradient method (APG),^39^ Singular Value Thresholding Algorithm (SVT),^40^ Augmented Lagrange multiplier method (ALM)^41^ and dual approach.^42^ In this work, we adopt the Augmented Lagrange Multiplier (ALM) method due to its efficiency. According to ALM, the Equation (9) can be converted to the following equivalent problem:

(10) $$\min_{R,N,J}{∥J∥}_{*}+\omega {∥N∥}_{2,1}s.t.\;D=DR+N,R=J$$

We further adopted Inexact ALM method to transform Equation (10) to an unconstraint problem, and then minimize this problem by utilizing augmented Lagrange function defined as follows:

(11) $$\begin{array}{cc} L(J,R,N)= & \;\;{∥J∥}_{*}+\omega {∥N∥}_{2,1}+\mathrm{tr}(Y_{1}^{T}(D-DR-N))+\\ & \mathrm{tr}(Y_{2}^{T}(R-J))+\frac{\mu }{2}\left({∥D-DR-N∥}_{F}^{2}+{∥R-J∥}_{F}^{2}\right) \end{array}$$

where μ > 0 is the penalty parameter. Equation (11) can be minimized with respect to *J*,* R* and *N*, respectively, by fixing the other variables and then updating the Lagrange multipliers *Y*_1_ and *Y*_2_. Concretely, we can fix the other variables to update *J* by the following rule:

(12) $$J=\arg\min\frac{1}{\mu }{∥J∥}_{*}+\frac{1}{2}{∥J-(R+Y_{2}/\mu )∥}_{F}^{2}$$

It is worth noting that Equation (12) has a closed‐form solution. It can be solved by Singular Value Thresholding (SVT) operator.^40^ Similarly, we can update *R* and *J* by fixing the others according to Equations (13) and (14) respectively:

(13) $$R={\left(I+D^{T}D\right)}^{-1}\left(D^{T}D-D^{T}N+J+\left(D^{T}Y_{1}-Y_{2}\right)/\mu \right)$$

(14) $$N=\arg\min\frac{\omega }{\mu }{∥N∥}_{2,1}+\frac{1}{2}{∥N-(D-DR+Y_{1}/\mu )∥}_{F}^{2}$$

Equation (14) can be solved by the following lemma^43^: Let *Q* be a given matrix, if the optimal solution to $\min_{W}\theta {∥W∥}_{2,1}+\frac{1}{2}{∥W-Q∥}_{F}^{2}$ is W*, then the *i*‐th column of W* is:

$${\left[W^{*}\right]}_{:,t}=\left\{\begin{array}{cc} \frac{{∥Q_{:,i}∥}_{2}-\theta }{{∥Q_{:,i}∥}_{2}}Q_{:,i}, & \text{if}{∥Q_{:,t}∥}_{2}>\theta \\ 0, & \text{otherwise} \end{array}\right.$$

After the *J*,* R*,* N* were updated, we could update the multipliers as follows:

(15) $$\begin{array}{cc} & Y_{1}=Y{}_{1}+\mu \left(D-DR-E\right)\\ & Y_{2}=Y_{2}+\mu \left(R-J\right) \end{array}$$

The convergence condition is ${∥D-DR-N∥}_{\infty }<\varepsilon$ and ${∥R-J∥}_{\infty }<\varepsilon$, where ɛ is a very small number (set as 1 × 10^−8^ in this paper). Finally, after the convergence condition is reached, we could get the pure data matrix R* and noise data matrix N* and then calculate a complete data matrix by *D* × R*. The procedure to solve Equation (9) is outlined in Algorithm 1. According to Algorithm 1, by replacing the input data matrix *D* with disease semantic similarity matrix *SD* as well as miRNA functional similarity matrix *FM*, we could obtain two refined similarity matrices *CD* and *CM* respectively.

**Table:** 

**Algorithm 1.** Solving Problem (9) by Inexact ALM
**Input**: Given an incomplete data matrix *D* and parameters ω ∈ (0, 1)
**Output**: complete matrix DR*
**Initialize**:* D* = 0, *E* = 0, *Y*_1_ = 0, *Y*_2_ = 0, μ = 10^−4^, max_μ_ = 10^10^, ρ = 1.1, ɛ = 10^−8^
**Repeat**: Fix the others and update ***J*** by: $\begin{array}{c} J=\arg\min\frac{1}{\mu }{∥J∥}_{*}+\frac{1}{2}{∥J-(R+Y_{2}/\mu )∥}_{F}^{2} \end{array}$ Fix the others and update ***R*** by: $R={\left(I+D^{T}D\right)}^{-1}\left(D^{T}D-D^{T}N+J+\left(D^{T}Y_{1}-Y_{2}\right)/\mu \right)$ Fix the others and update ***N*** by: $N=\arg\min\frac{\omega }{\mu }{∥N∥}_{2,1}+\frac{1}{2}{∥N-(D-DR+Y_{1}/\mu )∥}_{F}^{2}$ Update the multiplier *Y*_1_ and *Y*_2_ by: $\begin{array}{cc} & Y_{1}=Y{}_{1}+\mu \left(D-DR-E\right)\\ & Y_{2}=Y_{2}+\mu \left(R-J\right) \end{array}$ Update parameter μ by: $\mu =\min(\rho \mu ,\max_{\mu })$ Check the convergence condition by: ${∥D-DR-N∥}_{\infty }<\varepsilon$ and ${∥R-J∥}_{\infty }<\varepsilon$
**Until convergence**
**Return**: DR*

#### 2.5.2 Integration of similarity information

After *CD* and *CM* were obtained, we integrated them into existing similarity matrices as follows:

(16) $$FDS\left(i,\;j\right)=\left\{\begin{array}{cc} \frac{CD(i,\;j)+GD(i,\;j)}{2}, & \text{if}\;SD(i,\;j)=0\\ \frac{GD(i,\;j)+CD(i,\;j)+SD(i,\;j)}{3}, & \text{otherwise} \end{array}\right.$$

(17) $$FMS\left(i,\;j\right)=\left\{\begin{array}{cc} \frac{GM(i,\;j)+CM(i,\;j)}{2}, & \text{if}\;FM(i,\;j)=0\\ \frac{GM(i,\;j)+CM(i,\;j)+FM(i,\;j)}{3}, & \text{otherwise} \end{array}\right.$$

where *GD* and *GM* represent the Gaussian interaction profile kernel similarity for diseases and miRNAs respectively. Then, the final disease similarity matrix *FDS* and final miRNA similarity matrix *FMS* obtained by Equations (16) and (17) will be used to infer miRNA‐disease associations by label propagation.

#### 2.5.3 Label Propagation

Label propagation is a semi‐supervised learning method by propagating the labelled information to the unlabelled nodes iteratively in the whole network. For a specific disease *d*_*i*_, miRNAs that have interactions with this disease are considered as labelled samples (the corresponding entries in the *i*‐th row of matrix *DM* are 1s), while the other miRNAs are taken as unlabelled samples. Our objective is to uncover the potential associations between the unlabelled samples and the given disease by calculating the strength of their associations. Generally, a traditional label propagation problem can be defined as follows:

(18) $$Y^{t+1}=\alpha WY^{t-1}+\left(1-\alpha \right)I$$

where *t* is the time step and *Y* ^*t*+1^ represents the iteration results after *t* + 1 steps of label propagation. α∈[0, 1] is a hyper‐parameter which balanced the rate between retaining the information from its neighbours and its initial label information, *Y* is a binary matrix encoding the initial label information of data points against each class.^44^ Equation (18) has a closed‐form solution: *Y* = (1 − α)(*I* − α*L*)^−1^ *I*, where I is an identity matrix, *L* = *D* ^−1/2^ *WD* ^−1/2^ is the Laplacian matrix of *W* and *D* is the diagonal matrix with (*i*,* i*)‐th element equal to the sum of the *i*‐th row of *W*.^45^

Due to the high computational complexity induced by the matrix inversion operation of the closed‐form solution, we utilize Equation (18) to update the label of each data object until convergence. Therefore, we can predict miRNA‐disease associations from both disease space and miRNA space based on label propagation algorithm:

(19) $$F_{D}^{t+1}=\alpha \times FDS\times F_{D}^{t}+\left(1-\alpha \right)\times DM$$

(20) $$F_{M}^{t+1}=\alpha \times FMS\times F_{M}^{t}+\left(1-\alpha \right)\times DM^{T}$$

where *F*_*D*_ and *F*_*M*_ represent the prediction result from disease space and miRNA space respectively. The final association score is calculated by:

(21) $$F=\beta \left(F_{D}\right)+\left(1-\beta \right){\left(F_{M}\right)}^{T}$$

where β is a hyper‐parameter balancing the prediction results from disease space and miRNA space (β was simply set to 0.5 in this paper). The overall procedure of MCLPMDA is summarized in Algorithm 2. Besides, the source code of MCLPMDA can be freely downloaded at https://github.com/ShengPengYu/MCLPMDA.

**Table:** 

**Algorithm 2.** The Procedure of MCLPMDA
**Input**: Matrices *FM* ∈ ℝ^n*n^, *SD* ∈ ℝ^m*m^, *DM* ∈ ℝ^m*n^, parameter α and β.
**Output**: Predicted association matrix *F*.
1. Input *FM* to **Algorithm 1** and obtain the complete miRNA similarity matrix *CM*.
2. Input *SD* to **Algorithm 1** and obtain the complete miRNA similarity matrix *CD*.
3. Integrate similarity information to get *DSS* and *MFS* according to Equations (16) and (17).
4. Predict from miRNA space and disease space:
**Repeat**: $F_{D}^{t+1}=\alpha \times FDS\times F_{d}^{t}+\left(1-\alpha \right)\times DM$ $F_{M}^{t+1}=\alpha \times FMS\times F_{M}^{t}+\left(1-\alpha \right)\times DM^{T}$
**Until convergence**
5. Integrate the results $F=\beta \left(F_{D}\right)+\left(1-\beta \right)\times {\left(F_{M}\right)}^{T}$
6. **Return ** *F*

## 3 RESULTS

### 3.1 Performance evaluation

**Figure 2:**
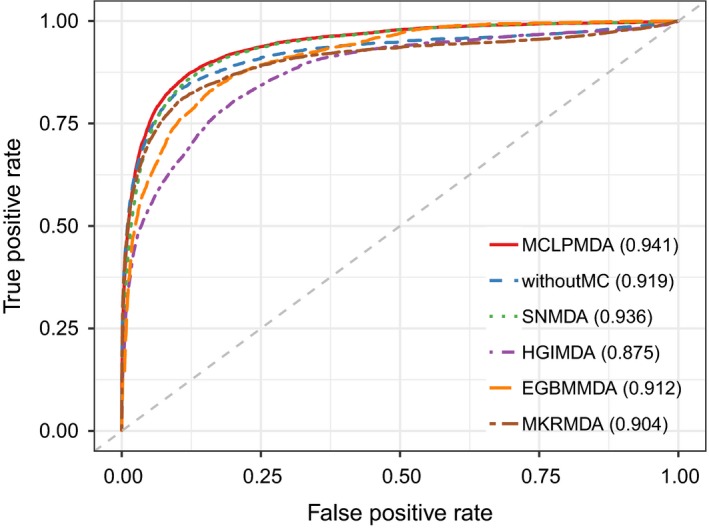
The comparison results between MCLPMDA and the other five methods in the framework of global LOOCV

**Figure 3:**
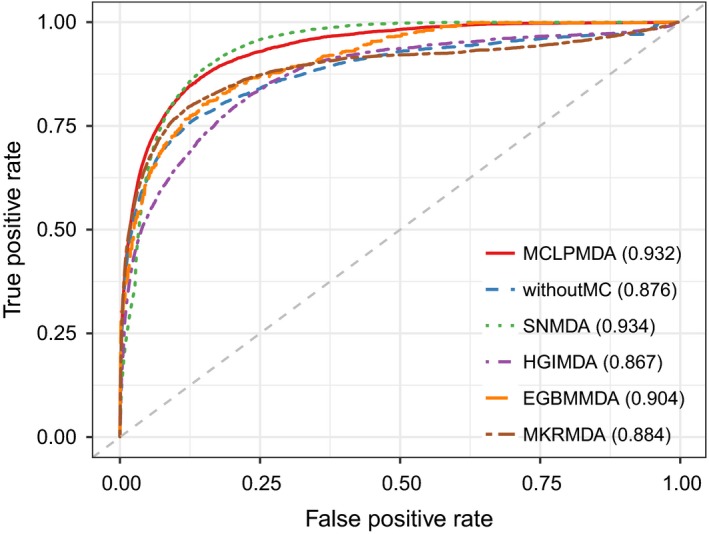
The comparison results between MCLPMDA and the other five methods in the framework of 5‐fold cross validation

In this section, we employed four different evaluation metrics to comprehensively evaluate the performance of MCLPMDA. We first implemented global LOOCV and 5‐fold cross validation to verify the general prediction ability of our method based on the experimentally verified miRNA‐disease associations from HMDD v2.0 databases.^31^ Specifically, global LOOCV selected a known miRNA‐disease association in turn as a test sample, and the rest of the associations were considered as training samples.^46^ As for 5‐fold cross validation, all known miRNA‐disease interactions were randomly divided into five groups, four of which were adopted as training samples and the remaining group was picked out as test samples. To avoid the bias caused by sample divisions, we repeated 5‐fold cross validation 100 times and used the average result of the 100 repetitions as the final output. In addition, the receiver operation characteristic curve was plotted by calculating the true positive rate and the false positive rate at varying thresholds to intuitively illustrate the prediction accuracy.^47^ The AUC value was then calculated to quantitatively evaluate the performance of MCLPMDA. Generally, the value of AUC ranges from 0 to 1 and the larger the AUC values the better the predicted results. As shown in Figures 2 and 3, MCLPMDA achieved reliable AUCs of 0.941 and 0.932 in global LOOCV and 5‐fold cross validation, respectively, which clearly demonstrated the favourable performance of our method. We further compared our method with four state‐of‐the‐art methods, that is, SNMDA, HGIMDA, EGBMMDA and MKRMDA. It is worth mentioning that SNMDA was also proposed by our team and achieved superior results.^48^ Moreover, in order to clearly demonstrate the power of our method, we removed the similarity matrices constructed by matrix completion for both miRNAs and diseases and compared its prediction performance with MCLPMDA in all cross‐validation frameworks. As a result, SNMDA, HGIMDA, EGBMMDA, MKRMDA and the method without matrix completion (without MC) obtained AUCs of 0.936, 0.875, 0.912, 0.904 and 0.919 in global LOOCV (Figure 2) respectively. In the framework of 5‐fold cross validation, they obtained AUCs of 0.934, 0.867, 0.904, 0.884 and 0.876 respectively (Figure 3). Although MCLPMDA is slightly less predictive than SNMDA in 5‐fold cross validation, it achieved the best performance in comparison with all the methods in global LOOCV.

**Figure 4:**
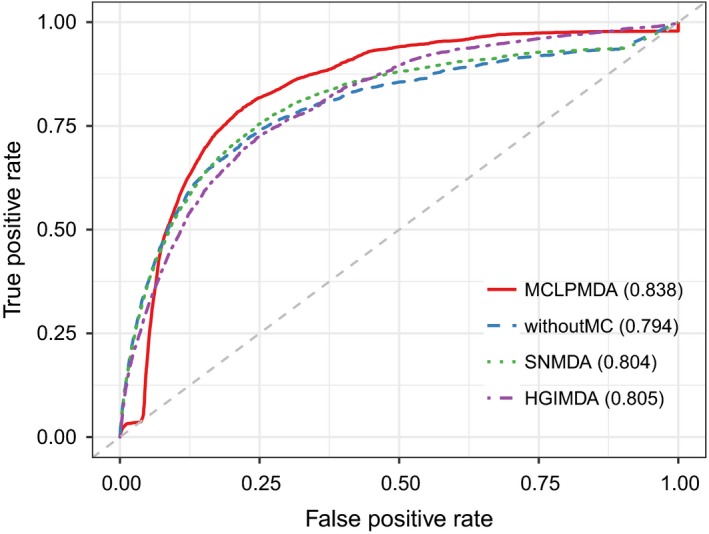
The comparison results between MCLPMDA and the other three computational models in terms of LODOCV

Next, we adopted another evaluation metric called leave one disease out cross validation (LODOCV) to test the ability of our method to predict for diseases without any known associated miRNAs. Specifically, for each disease, we removed all its associated miRNAs and then prioritized all the candidate miRNAs using the information of other disease‐related miRNAs. As there is no prior association information for the disease investigated, LODOCV is considerably more stringent compared with the cross‐validation frameworks mentioned above and can thus better evaluate the risk of overfitting. Finally, AUC value was used to evaluate the performance of all methods in LODOCV framework. As shown in Figure 4, MCLPMDA achieved the highest AUC value of 0.838 in LODOCV framework among all methods. We did not demonstrate the performances of EGBMMDA and MKRMDA in the figure as the AUC values obtained by both methods were lower than 0.5. Additionally, we calculated the statistical significance of performance improvement gained by MCLPMDA over the other methods to clarify the efficacy of our method. Concretely, we computed an AUC value for each disease and obtained a vector consisting of 383 AUC values for each method. We then assessed the statistical significance of difference between AUC values of different methods by Wilcoxon signed rank test. As shown in Table 1, our method significantly improved the prediction performance with respect to the other four methods in LODOCV, which clearly confirms the generalization ability of MCLPMDA in predicting new miRNA‐disease associations.

**Table 1:** Statistical significance of differences in performance between the proposed method and the other five methods in LODOCV. *P*‐values were calculated by Wilcoxon signed rank test

	SNMDA	HGIMDA	EGBMMDA	MKRMDA	Without MC
*P*‐value	3.67e‐02	4.24e‐04	4.54e‐98	2.12e‐41	1.95e‐03

**Figure 5:**
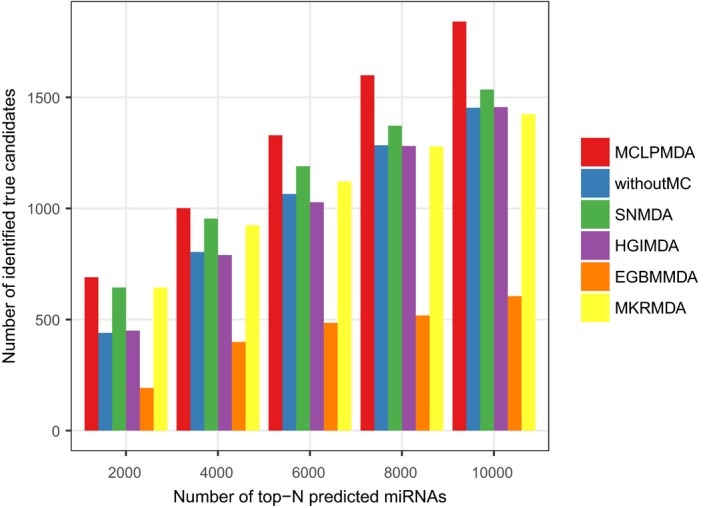
The number of true miRNA‐disease associations identified by each method

Finally, to further demonstrate the real discovery ability of our method, we applied our model on the older version of HMDD (v1.0) and then validated the predicted miRNA‐disease associations by the latest version of HMDD (v2.0). Specifically, there were 1036 known associations involving 221 miRNAs and 122 diseases recorded in HMDD v1.0 after filtering. For each method, we selected the top‐*N* predicted miRNAs with *N* ranging from 2000 to 10 000 with an interval of 2000 and then counted the number of identified true candidates recorded in HMDD v2.0. As clearly demonstrated in Figure 5, MCLPMDA could also identify more disease‐associated miRNAs than the other four computational alternatives. Taken together, the various validation results verified the superior performance of our methods in predicting potential associations between miRNAs and diseases.

### 3.2 Case study

In this section, we conducted a case study on BN to further validate the effectiveness of MCLPMDA. BN is the most common malignancy in women, accounting for >40 000 deaths each year.^49^ Data have shown that the number of affected people is climbing, and a forecast deemed that there will be nearly 3.2 million new patients per year by 2050.^50^ Researchers have found that many miRNAs are associated with BN by clinical experiments, such as mir‐155 and mir‐21, both of which can lead to BN tumourigenesis or metastasis.^51^ For the investigated disease, we listed the top 50 miRNAs prioritized by our method based on the known miRNA‐disease associations from HMDD v2.0. The prediction results were verified by another two databases dbDEMC^52^ and miR2Disease,^53^ both of which record experimentally verified disease‐related miRNAs. Our prediction results showed that 10 of the top 10 and 49 of the top 50 candidate miRNAs were verified to be associated with BN by at least one of the two databases. As shown in Table 2, only hsa‐mir‐449a was not confirmed by our method. As a matter of fact, the study of Shi et al. has shown that hsa‐mir‐449a was implicated functionally in breast cancer pathogenesis by suppressing Cysteine‐Rich Protein 2 and altering cell viability, migration, invasion, in vivo tumour growth and angiogenesis, thereby driving malignant phenotypes in these aggressive tumours.^54^

**Table 2:** Top 50 predicted miRNAs associated with Breast Neoplasms based on known associations in HMDD. The first column records the top 1‐50 predicted miRNAs; the second column records the corresponding evidence in two databases; the third column records log2 fold change and the fourth column records the adjusted *P*‐values of the significance of differential expression for each miRNA

miRNA	Evidence	logFC	FDR
hsa‐mir‐125a	dbDEMC;miR2Disease	−0.50551	6.02e‐12
hsa‐mir‐196a	dbDEMC;miR2Disease	3.210542	1.04e‐37
hsa‐mir‐499a	dbDEMC	−1.8022	4.51e‐26
hsa‐mir‐198	dbDEMC;miR2Disease	−0.60211	3.93e‐02
hsa‐let‐7a	dbDEMC;miR2Disease	−0.17426	9.69e‐02
hsa‐mir‐141	dbDEMC	2.216414	4.65e‐74
hsa‐mir‐143	dbDEMC;miR2Disease	−1.17215	1.97e‐28
hsa‐mir‐145	dbDEMC;miR2Disease	−2.37613	3.00e‐224
hsa‐mir‐150	dbDEMC	−0.05109	1.00e+00
hsa‐mir‐16	dbDEMC	0.394382	4.28e‐05
hsa‐mir‐21	dbDEMC	2.143077	1.52e‐110
hsa‐mir‐1	dbDEMC	−5.68257	8.66e‐254
hsa‐mir‐133a	dbDEMC;miR2Disease	−6.50194	0.00e+00
hsa‐mir‐133b	dbDEMC;miR2Disease	−6.68341	2.74e‐190
hsa‐mir‐146a	dbDEMC	0.501373	1.37e‐04
hsa‐mir‐208b	dbDEMC;miR2Disease	−4.35801	2.98e‐62
hsa‐mir‐103a	dbDEMC	0.809716	1.35e‐15
hsa‐mir‐106a	dbDEMC;miR2Disease	0.999651	3.51e‐12
hsa‐mir‐10b	dbDEMC;miR2Disease	−1.88876	1.34e‐94
hsa‐mir‐126	dbDEMC;miR2Disease	−0.98217	9.66e‐36
hsa‐mir‐135a	dbDEMC;miR2Disease	1.217938	1.75e‐03
hsa‐mir‐151a	dbDEMC;miR2Disease	0.417736	2.23e‐07
hsa‐mir‐152	dbDEMC	−0.15395	1.38e‐01
hsa‐mir‐181b	dbDEMC;miR2Disease	1.397101	8.49e‐30
hsa‐mir‐182	dbDEMC;miR2Disease	2.364107	2.39e‐63
hsa‐mir‐183	dbDEMC	2.946886	1.06e‐95
hsa‐mir‐191	dbDEMC;miR2Disease	1.217488	2.41e‐29
hsa‐mir‐192	dbDEMC	1.468736	2.60e‐37
hsa‐mir‐193b	dbDEMC	−0.02624	1.00e+00
hsa‐mir‐194	dbDEMC;miR2Disease	0.496013	2.49e‐07
hsa‐mir‐200a	dbDEMC;miR2Disease	2.10741	1.56e‐64
hsa‐mir‐200b	dbDEMC;miR2Disease	1.698791	6.59e‐41
hsa‐mir‐200c	dbDEMC	1.53758	2.83e‐44
hsa‐mir‐203	dbDEMC	2.262136	6.25e‐23
hsa‐mir‐204	dbDEMC;miR2Disease	−2.62831	2.42e‐62
hsa‐mir‐205	miR2Disease	−1.46212	2.66e‐19
hsa‐mir‐20a	dbDEMC	0.784424	1.26e‐09
hsa‐mir‐210	dbDEMC	3.06042	6.75e‐48
hsa‐mir‐215	dbDEMC	−1.27642	5.39e‐28
hsa‐mir‐221	dbDEMC	−0.07311	7.72e‐01
hsa‐mir‐223	dbDEMC	−0.8271	7.04e‐13
hsa‐mir‐25	dbDEMC;miR2Disease	−0.0555	7.34e‐01
hsa‐mir‐26b	dbDEMC	−0.22093	7.48e‐03
hsa‐mir‐31	dbDEMC;miR2Disease	0.25524	3.00e‐01
hsa‐mir‐34b	dbDEMC	0.253323	2.36e‐01
hsa‐mir‐429	dbDEMC	2.689754	5.04e‐72
hsa‐mir‐449a	Unconfirmed	5.627081	2.54e‐25
hsa‐mir‐449b	dbDEMC	4.278504	1.73e‐17
hsa‐mir‐92a	dbDEMC	−0.27138	5.93e‐03
hsa‐mir‐93	dbDEMC	1.137218	1.98e‐29

**Figure 6:**
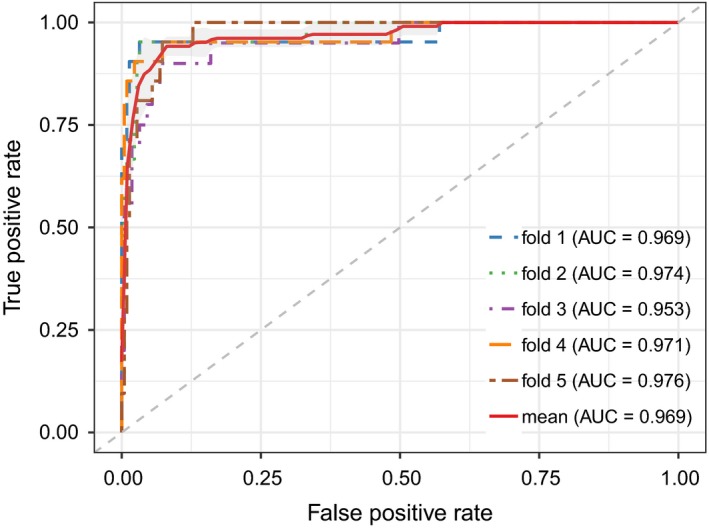
The prediction accuracy of 5‐fold cross validation classification of tumour samples based on the top 6 prioritized miRNAs

To further verify the diagnostic power of the top prioritized miRNAs, we downloaded miRNA expression data as well as the corresponding clinical information of real patients from The Cancer Genome Atlas ( https://portal.gdc.cancer.gov/repository) for BN. Specifically, the downloaded miRNA expression data contains 104 normal samples and 1096 tumour samples involving 1881 miRNAs. To carry out a thorough analysis towards the top predicted miRNAs, we first calculated the differentially expressed miRNAs by using the R package edgeR.^55^ Concretely, edgeR automatically calculates the log2 fold change and the statistical significance of differential expression of each miRNA. It also provides the adjusted *P*‐values for multiple testing correction with false discovery rate (FDR). As a result, 29 of the 50 miRNAs were differentially expressed (adjusted *P*‐value <0.05 and |logFC| >1, Table 2). We then tested whether these top predicted miRNAs could be used as features to classify normal samples and tumour samples. Support vector machine from R package e1071 was adopted to perform the classification analysis. The radial basis function was chosen as the kernel function, and the best values of the two parameters *cost* (C) and *gamma* (γ) in the kernel function were obtained by a grid‐search approach using cross‐validation. Finally, the classification accuracy was evaluated by five‐fold cross‐validation. We found that the top 6 miRNAs could achieve a mean classification accuracy of 0.969 (Figure 6), which clearly demonstrates the classification power of the top prioritized miRNAs.

**Figure 7:**
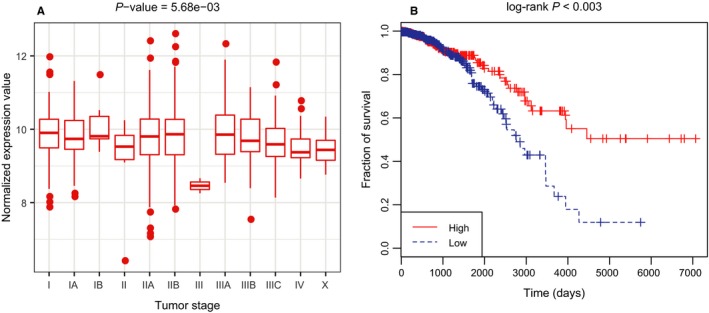
(A) The expression levels of hsa‐mir‐125a at different pathologic stages; (B) Kaplan–Meier survival analysis for hsa‐mir‐125a. As observed, patients with higher expression level are at lower risk level

Next, we focused on hsa‐mir‐125a, the top predicted miRNA for BN. We checked whether its expression level was significantly altered at different tumour stages by one‐way ANOVA test. Only tumour samples were retained and there were in total 1067 tumour samples with clinical information after matching the patient barcode with sample names. After filtering, there are 12 pathologic stages in patients, i.e. Stage I, IA, IB, II, IIA, IIB, III, IIIA, IIIB, IIIC, IV and X. The one‐way ANOVA test was performed by the R built‐in function “aov.” As a result, we obtained a *P*‐value of 5.68e‐3 (Figure 7A), indicating that its expression level was significantly altered among different stages. Besides, we performed the Kaplan–Meier survival analysis to examine its potential diagnostic power by using the R package survival. Notably, different expression levels of hsa‐mir‐125a have led to significantly different survival rate (Figure 7B). Taken together, the analysis results verified that hsa‐mir‐125a could serve as a potential biomarker for BN.

## 4 DISCUSSION

Traditional experimental methods are in general time‐consuming and cannot be scaled to large datasets. Fortunately, the accumulating amount of data from multiple sources have posed great opportunities to identify miRNA‐disease associations computationally at a large scale. In this study, we proposed a novel computational model to predict the underlying miRNA‐disease associations based on matrix completion and label propagation. Considering the sparsity and incompleteness of disease semantic similarity and miRNA functional similarity matrix, we first used the matrix completion algorithm to obtain refined similarity matrices and then combined them with existing similarity information. To demonstrate the effectiveness of MCLPMDA, we applied different evaluation metrics to measure the prediction performance and the experimental results demonstrated the utility of our method. We then compared MCLPMDA with four state‐of‐the art methods and the comparison results further confirmed the superior performance of MCLPMDA. Lastly, the case study conducted on BN also validated the prediction ability of MCLPMDA. Notably, our method could be applied to diseases without any known related miRNAs, which is often the case in practical use. In summary, all these results indicated that MCLPMDA can effectively uncover new disease‐related miRNAs.

The success of our model could be mainly attributed to the following two reasons. First, matrix completion was adopted to refine the miRNA functional similarity matrix and disease semantic similarity matrix, which greatly alleviated the influences caused by the inherent noise existing in the current datasets. Second, the label propagation process ensured that the labels of candidate miRNAs were reliably updated based on the reconstructed similarity matrices. Nevertheless, the performance of our model can still be improved. In particular, more data sources such as miRNA target information and miRNA sequence similarities could be incorporated to further elevate the prediction accuracy. Besides, adaptive weights should be assigned instead of equal weights when combining the refined similarity matrices with existing similarity information for both miRNAs and diseases.

## CONFLICT OF INTEREST

The authors confirm that there are no conflicts of interest.
